# Binary Diffusion Coefficients for Short Chain Alcohols in Supercritical Carbon Dioxide—Experimental and Predictive Correlations

**DOI:** 10.3390/molecules28020782

**Published:** 2023-01-12

**Authors:** Cecília I. A. V. Santos, Ana C. F. Ribeiro, Valentina Shevtsova

**Affiliations:** 1 CQC-IMS, Department of Chemistry, University of Coimbra, 3004-535 Coimbra, Portugal; 2 Mechanical and Manufacturing Department, Mondragon University, 20500 Arrasate-Mondragón, Spain; 3 Ikerbasque, Basque Foundation for Science, 48011 Bilbao, Spain

**Keywords:** supercritical carbon dioxide, FTIR, Taylor dispersion technique, methanol, ethanol, propanol, butanol, Widom line

## Abstract

Experimental binary diffusion coefficients for short-chain alcohols in supercritical carbon dioxide were measured using the Taylor dispersion technique in a temperature range of 306.15 K to 331.15 K and along the 10.5 MPa isobar. The obtained diffusion coefficients were in the order of 10^−8^ m^2^ s^−1^. The dependence of *D* on temperature and solvent density was examined together with the influence of molecular size. Some classic correlation models based on the hydrodynamic and free volume theory were used to estimate the diffusion coefficients in supercritical carbon dioxide. Predicted values were generally overestimated in comparison with experimental ones and correlations were shown to be valid only in high-density regions.

## 1. Introduction

Research on supercritical fluids (SCFs) science and technology has advanced in the past decades at both academic and industrial levels due to the broad applications of SCFs. Supercritical technologies are employed in a multitude of areas such as food, cosmetics and pharmaceuticals by replacing traditional organic solvents [1,2] oil industries (for biodiesel preparation purposes [3]) or working fluid (for shale gas recovery [4]). More recently, new technologies have been developed to take advantage of SCFs’ excellent heat transfer properties, employing them as heat transfer fluids, e.g., in heat and power plants [5], or in the development of new geothermal energy systems [6,7], as they have the advantage of being environmentally friendly when compared with conventional methods that pollute or contribute to the greenhouse effect. Still within the topic of global warming, supercritical technologies have been specially investigated as a valid approach to mitigating climate change, namely through carbon capture and storage (CCS) mechanisms [8,9]. Carbon dioxide, amidst supercritical fluids, has been largely used due to its chemical stability, substantial inertness, low critical temperature, relatively non-toxicity, non-flammability and availability in high purity at a relatively low cost. Above its supercritical point, (*p_c_* = 7.38 MPa, *T*_c_ = 304.18 K) carbon dioxide becomes a fluid (scCO_2_) that can expand like a gas but has the density of a liquid [10,11]. This low viscosity, combined with its high solute diffusivity gives it superior solvating properties and mass transfer characteristics in comparison with more conventional solvents (e.g., liquids). Indeed, these features, together with the ability to solvate numerous polar and non-polar compounds, justify the green solvent label given to carbon dioxide and its large range of applications.

While it is relatively easy to find literature sources with data on phase equilibria and/or solubilities of diverse compounds in supercritical CO_2_ [12,13,14], information on their transport properties is scarce. Transport properties, together with solubility data, are necessary information for the design of processes wherein mass transfer is the dominant rate mechanism, such as chemical reactors or process development and efficiency improvement of supercritical fluid extraction. Lack of fundamental thermodynamic data required for process design and scale up also restrains the development of supercritical fluid technologies up to an acceptable commercial level of performance. Hence, binary diffusion coefficients are parameters needed in almost all useful models for multicomponent diffusivity. They are also of critical importance to properly illustrate transport properties due to the reduced number of models and the limited conditions in which the existent theoretical and empirical models, capable of describing diffusion, are valid. Moreover, since most real systems are multicomponent, knowledge of mass transfer rates and understanding of the transport processes of solutes in supercritical carbon dioxide justify themselves in order to comprehend the role that individual and overall mass transfer coefficients play as intrinsic contributors to multicomponent diffusivities.

Model systems such as supercritical carbon dioxide combined with short chain alcohols, are important in the pharmaceutical and food industries, namely in supercritical fluid extraction and supercritical fluid chromatography processes [12,15,16,17], where alcohols act as modifiers (cosolvents) to improve the selectivity and efficiency of the CO_2_ extraction process. These scCO_2_+alcohols-model systems are also of high significance in the petroleum and natural gas industries, where methanol and ethanol may act as inhibitors to prevent hydrate plug formation on wells, in the Rectisol process and in the production of alcohols from syngas [8,9,10]. In this sense, quantitative knowledge of rate mechanisms, such as binary molecular diffusion of alcohols in supercritical carbon dioxide, is crucial for equipment design, process development and efficient operation of supercritical fluid extraction, but also for the improvement of empirical models so that it becomes possible to accurately predict properties in multicomponent mixtures.

We present results for the experimental measurements of binary diffusion coefficients of several short chain alcohols, e.g., methanol, ethanol, 1-propanol, 2-propanol and 1-butanol, in scCO_2_, covering a wide density range both close and far from the critical point in order to understand the transport phenomena occurring in those systems. Classical empirical correlations are also used to estimate the diffusion coefficients and the predicted results and experimental data are compared.

## 2. Results

### 2.1. Experimental Diffusion Coefficients for Alcohols in Supercritical CO_2_

Diffusion coefficients for short chain alcohols, methanol, ethanol, 1-propanol, 2-propanol, and 1-butanol, in supercritical carbon dioxide, at a pressure of 10.5 MPa and at temperatures ranging from 306.15 to 331.15 K, are presented in Table 1. Values for density and viscosity [18] for supercritical carbon dioxide in the same range of temperatures are also presented. The diffusion coefficients were obtained from an average of at least six replicate dispersion profiles, and the standard deviation was estimated from the averaged diffusion coefficients over different wavenumbers for each run, over repeated runs.

### 2.2. Predicted Diffusion Coefficients for Alcohols in Supercritical CO_2_

Given the need for accurate data on the transport properties of solutes in scCO_2_ and the impracticability of experimentally measuring all the systems of interest, at all operation conditions, it is important to be able to rely on models that provide a consistent estimation of the diffusion coefficients. Molecular dynamics simulations are an effective alternative but require some experimental data to establish MD parameters [20]. Great effort has been devoted to creating expressions valid for predicting their diffusion in supercritical fluids, and the literature is rich in empirical models for their prediction. They can be commonly classified in two groups: models constructed on the base of rough hard sphere principles [21,22,23] and hydrodynamic models based on the Stokes–Einstein equation [24,25,26,27,28,29,30]. A big number of these models present some limitations, failing in their estimations of *D* for polar or asymmetric solutes, or cannot be applied over a wide range of temperatures and pressures, nor near the critical point.

Here, we have chosen to test our experimental data against the improved Stokes –Einstein models developed by Vaz et al. [29], where universal parameters valid for scCO_2_ were introduced in the original hydrodynamic correlations developed by Wilke-Chang [25], Scheibel [31] and Lusis–Ratcliff [32]. We also test predictive equations from Sassiat [24] and Lai–Tan [28]’s models, developed and /or validated for solutes diffusing in supercritical carbon dioxide, and He and Yu [23]’s model based on free volume theory.

In general, these correlations have specific constants obtained from the fitting of a relatively large database of different solutes, and introduce correction parameters such as solvent viscosity and solute molar volume or molecular weight. While the classical models such as Wilke–Chang, Scheibel and Lusis–Ratcliff, originally developed for diffusion in liquids, are expected to fail in predictions of diffusion coefficients in regions of low viscosity (as the original Stokes–Einstein equation does), the modified equations by Vaz et al., together with Sassiat, Lai–Tan and He and Yu models, built for solutes in supercritical carbon dioxide, should be able to provide more accurate predictions.

The ability of the above-mentioned models to predict diffusion coefficients, when compared with the experimental ones, is evaluated in terms of the average absolute deviations (*AAD%*), calculated from:

(1) $$AAD\;\left(\%\right)=\frac{100}{n}\overset{n}{\underset{i=1}{\sum }}{\left|\frac{D_{exp}-D_{calc}}{D_{exp}}\right|}_{i}$$

where the subscripts “*exp*” and “*calc*” refer to the experimental and calculated diffusion coefficients, respectively, and *n* is the number of experimental points.

The results obtained for the various correlations tested for the prediction of diffusion coefficients of methanol, ethanol, 1-propanol, 2-propanol and 1-butanol in supercritical carbon dioxide are shown in Table 2.

## 3. Discussion

Historically, CO_2_ has been treated as a nonpolar solvent, comparable to alkanes due to its low dielectric constant and dipole moment, whereas recent studies suggest that it has the potential to act as both a weak Lewis acid and Lewis base [33]. Additionally, strong theoretical and experimental evidence indicates that CO_2_ can participate in conventional and nonconventional hydrogen-bonding interactions. For example, experimental investigations reveal the formation of conventional hydrogen bonds between an oxygen atom of CO_2_ and hydroxyl (–OH) groups [34]. These site-specific solute–solvent interactions may help to understand the fundamental nature of scCO_2_ as a solvent and contribute to the interpretation of our experimental results.

**Figure 1:**
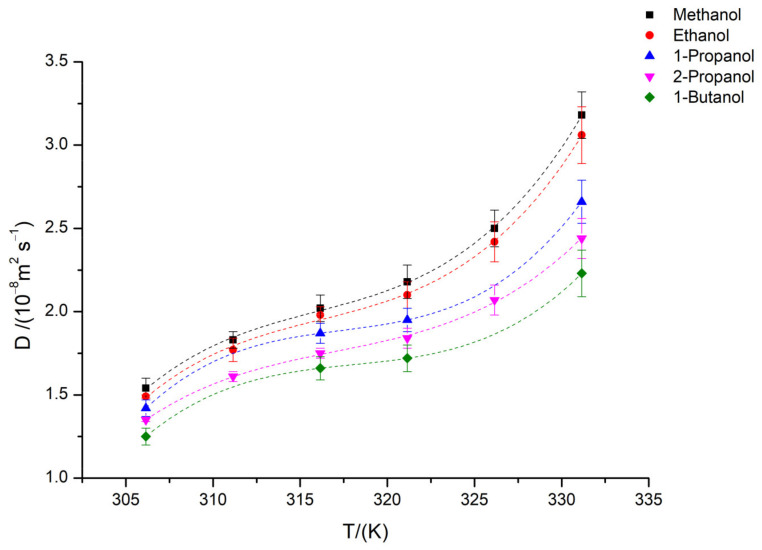
Diffusion coefficient for methanol, ethanol, 1-propanol, 2-propanol, and 1-butanol in scCO_2_ at *p* = 10.5 MPa and at different temperatures from 306.15 to 331.15 K. Dots are experimental values and dashed lines the fitting.

The diffusion coefficients for alcohols in supercritical CO_2_, illustrated in Figure 1, show a marked increase as the temperature rises, probably related but not limited to the increase in the kinetic energy of the molecules. Alcohols with higher molecular weight appear to be less affected by the temperature increase. Indeed, the heavier alcohol 1-butanol shows an increase of 56% in the mobility over a 25 K temperature interval, whilst the diffusion coefficient for the lighter alcohol methanol increases up to 100%.

The hydrogen bonding between the CO_2_ and OH groups, in the particular case of 1-propanol and 2-propanol, gives rise to different structures; they are linear and spherical structures, respectively, suggesting differences in the solute due to exposure to the solvent. The solute–CO_2_ interactions are favored over the solute–solute ones in promoting the electrostrictive effect of the CO_2_ molecules (that is, causing the solvent to contract), the effect being more significant in the case of 1-propanol. This statement is also in agreement with the results presented in Table 1 for the diffusion of these alcohols. In fact, when comparing isomers 1-propanol and 2-propanol, the latter is seen to diffuse slower than the linear molecule, with approximately a 10% decrease in the diffusion coefficient. If one considers the molecule’s shape, 2-propanol is branched and thus a more spherical molecule, with a slightly bigger molar volume, indicating lesser electrostriction of CO_2_ molecules by this alcohol when compared with the other isomer. The entities of this alcohol offer more frictional resistance to motion through the scCO_2_ and, consequently, the alcohol’s diffusion coefficient is minor. This observation is in agreement with other studies that found that linear molecules generally tend to diffuse faster than spherical molecules of the same molar volume, both in liquids [35] and supercritical fluids [36].

Another interesting observation is that the relationship between the diffusion coefficient and temperature for these solutes is non-linear, and the coefficients for polynomial fit are presented in Table 3. Previous studies have shown that this non-linear relationship, with an inversion of the slope in the region where the mobility is the maximum for scCO_2_, can be explained by the presence of the Widom line [10,37]. The presence of this transition zone, contrary to a simple homogeneous supercritical state space, splits the phase diagram into two supercritical regions: liquid-like and gas-like. The latter are considered extensions of the subcritical liquid and gas phases [38,39,40], where supercritical fluid properties continuously change over a narrow temperature range. Widom line presence has been verified by experimental and theoretical studies [39], and the crossing of this region is associated with a minimum value of the thermodynamic factor, this behavior being explained by the free volume increment associated with density variation [41], thus affecting the transport properties of supercritical carbon dioxide. Interpretations and definitions of the Widom line are still subject of some debate. A general principle estimates the Widom line from the locus of maxima of different thermodynamic response functions that originate at the critical point [42]. Since different functions give rise to slightly distinct lines, sometimes these changes are referred also as the Widom region. When based on transport properties, the separation between liquid and gas-like regions is generally defined by the minimum of the kinematic viscosity [43].

The maximum of mobility (in terms of the inverse of the kinematic viscosity) for pure CO_2_ at 10.5 MPa and the inflection point for density curves, calculated from the data obtained from NIST [18], take place at around T ≈ 322 K. This is also in accordance with isobaric MD simulations [39], experimental results obtained for CO_2_ [44] and similar studies in the literature [10,19,37,40]. Inflection points obtained from the fitting of the dependence of the diffusion coefficients with temperature, for each of the systems studied here, are observed at temperatures between 319 K (for the smaller molecule methanol) and 325 K (for butanol). This small shift in the temperature where the transport properties change can be related to the molecular interactions occurring in the solution. For methanol, it may be the result of strong hydrogen bonding between the solute and the solvent giving rise to a more ordered and less dense diffusion media for the methanol molecules to diffuse in; meanwhile, for the bulkier molecules, it is possible that the increased viscosity of the mixture, in contrast to a smaller viscosity of the pure CO_2_, plays an important role even in this range of very dilute solutions.

**Table 3:** Coefficients for third order polynomial fitting of the temperature dependance of the diffusion coefficients for short chain alcohols in supercritical carbon dioxide.

Solute	*A*	*B*	10^2^ *C*	10^4^ *D*	R^2^	Inflection Point
Methanol	−6707	63.66	−20.01	2.10	0.999	319
Ethanol	−6601	62.60	−19.79	2.04	0.997	323
1-Propanol	−7162	67.70	−21.30	2.20	0.997	323
2-Propanol	−4421	41.79	−13.01	1.34	0.998	324
1-Butanol	−5835	55.03	−17.30	1.77	0.998	325

**Figure 2:**
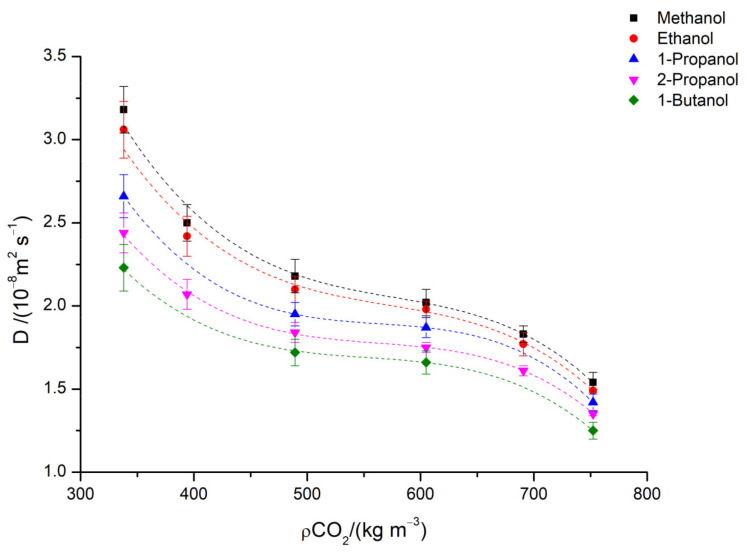
Diffusion coefficient for methanol, ethanol, 1-propanol, 2-propanol and 1-butanol in scCO_2_ at *p* = 10.5 MPa and at different temperatures from 306.15 to 331.15 K. Dots are experimental values and dashed lines are the fitting.

When analyzing the diffusion coefficients *D* for a determined solute in carbon dioxide, it is common to use density instead of the operating temperature and pressure, since these two parameters define supercritical carbon dioxide density and viscosity. Figure 2 shows the behavior of the diffusion coefficients for the short chain alcohols here studied in supercritical CO_2_ in isobaric conditions (a pressure of 10.5 MPa and temperatures ranging from 306.15 to 331.15 K)**.** The values of density for supercritical carbon dioxide were obtained from NIST [18].

The diffusion coefficient for alcohols in scCO_2_ decreases with the increasing density of supercritical CO_2,_ and the reason for this decrease is related to the larger number of molecular collisions and the reduced mean free path accessible for the molecules. As the density increases, solvent molecules are closer, and this restricts the movement of solute molecules in the media.

**Figure 3:**
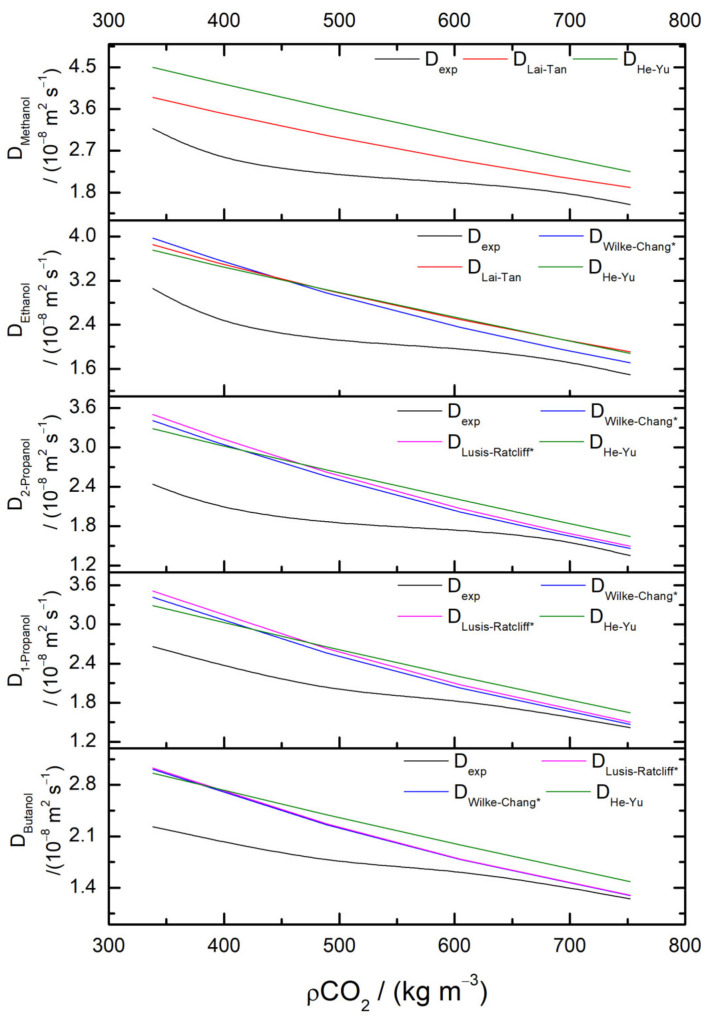
Experimental and predicted diffusion coefficients for methanol, ethanol, 1-propanol, 2-propanol, and 1-butanol in scCO_2_ at pressure *p* = 10.5 MPa and at temperatures from 306.15 to 331.15 K.

Empirical correlations have been tested to verify their ability to correctly predict the experimental values found here. The empirical equations used were based in hydrodynamic and free volume theory. Figure 3 presents some of the predictive curves for each of the short chain alcohols under study, and for the models which overall showed a better agreement with experimental data.

In general, none of the equations was able to predict the behavior of the diffusion coefficients determined experimentally over the complete density range. Predictive curves for the diffusion coefficients were overestimated, even by the models developed for diffusion in supercritical carbon dioxide. Notwithstanding, AAD% is generally low due to the high number of experimental runs; the relative deviations from experimental data in the lowest density regions can go up to 100%, and estimation is poor independent of the size of the molecules, even if the majority of the equations contemplate a parameter accounting for the size of the solute. 

Yet, for the higher densities of scCO_2_ (> 600 Kg m^−3^), all models, independent of the theory they are based on, can provide a reasonable estimation of *D* (between 3 and 20% deviation), which is a good approximation if we also take into account the uncertainties associated with the experimental measurements. Modified equations by Vaz et al. [29] for Wilke and Chang and Lusis–Ratcliff hydrodynamic correlations, built based on the dependance of *D* on the solvent viscosity and incorporating a parameter based on the size of the solute (molar volume), together with He and Yu model, show the best agreement with experimental data. Finally, in this range of densities, an increase in the molecular size (molecular weight and molar volume) produces closer predictions to the experimental results, probably because most of the models were developed and tested for solutes of a relatively large size. In conclusion, they can be assumed to be reliable models for the calculation of the diffusion coefficients of solutes in supercritical CO_2_, particularly for new unknown systems at any condition in which information is completely absent.

## 4. Materials and Methods

### 4.1. Materials

Methanol 99.99% (CAS Number: 67-56-1) was supplied by Fisher Scientific, ethanol absolute 99.97% (CAS Number: 64-17-5) was supplied by VWR Chemicals Prolabo, 1-propanol >99.5% (CAS Number: 71-23-8) was supplied by TCI Europe, 2-propanol pro analisis >99.8% (CAS Number: 67-63-0) was supplied by Sigma-Aldrich and 1-butanol >99.8% (CAS Number: 71-36-3) was supplied by RCI Labscan. Each liquid was used as received, with no further purification. CO_2_ with purity higher than 99.995% (water content <40 ppm) was supplied by Air Liquide.

### 4.2. Equipment and Experimental Procedure

**Figure 4:**
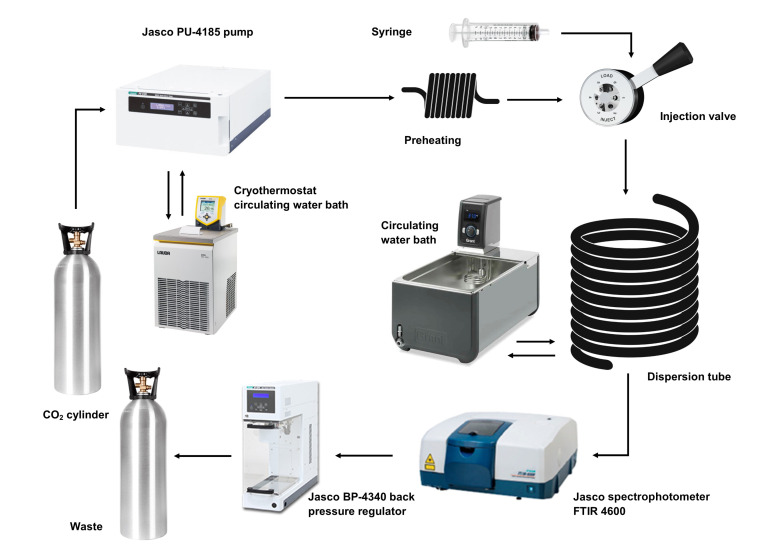
Schematic representation of the high-pressure Taylor dispersion set-up.

The Taylor dispersion technique (TDT) here employed has been optimized for the measurement of high pressure and supercritical fluids [19,45], namely scCO_2_, and is represented in Figure 4. It operates upon the fundamental basis of TDT: a pulse of a solute that is injected into a solvent stream, flowing under a laminar regime through a circular cross section capillary tube, will widen due to the joint action of convection occurring in the longitudinal axis and molecular diffusion occurring in the radial direction. Diffusion studies in supercritical CO_2_ are conditioned by the critical parameters of carbon dioxide, temperature and pressure, that is, *T*_c_ = 304.18K and *p*_c_ = 7.38MPa [46]. Determination of the diffusion coefficients is achieved by injecting, in the start of each experiment, a pulse of 5 μL of solute through a 6-port injection valve (Knauer model A1357, Knauer, Berlin, Germany) into CO_2_ at a constant stream rate of 0.3 cm^3^ min^−1^, maintained by a HPLC analytical pump (Jasco PU-4185, Jasco Inc., Hachioji, Japan). The pump head has a custom designed cooling device attached to it; temperature is regulated by a Peltier module controlled by a circulating water bath set to 260.15 K (Lauda Eco RE415G, Lauda, Lauda-Königshofen, Germany). This cooling device is aimed to keep CO_2_ in the liquid state, allowing for the pump to pressurize it above its critical pressure into the diffusion tube. A heat exchanger of 1.5 m long is placed at the pump outlet and used for preheating subcooled liquid CO_2_ to its supercritical state before the injection valve. The diffusion tube consists of a stainless-steel capillary tube of (30.916 ± 0.001) m length and 0.375 mm inner radii. It is coiled on a channeled aluminum cylinder in the form of a helix with 0.36 m diameter for both support and temperature regulation and kept at the study temperature ±0.1 K using a temperature-regulated water bath (Grant GD100, Grant Instruments Keison, Chelmsford, UK). Dispersion of the injected samples is monitored using a FT-IR refractometer (Jasco FT-IR 4600, Jasco Inc.), placed at the outlet of the dispersion tube and equipped with a high-pressure demountable cell (Harrick, Pleasantville, NY, USA), optimized for the best possible signal-to-noise ratio [45]. The detector is connected to a computer for digital data acquisition (Spectra Manager v.2 software by Jasco) allowing it to follow the response curves corresponding to the changes in the stream with time and in terms of absorbance/transmittance infrared spectra at wavenumbers corresponding to the different vibration modes of the molecules studied here. The pressure in the system is controlled by a back pressure regulator (Jasco BP-4340, Jasco Inc.) together with a pressure sensor (Jumo dTrans p30, JUMO Process Control, Inc., East Syracuse, NY, USA) for maintaining ± 0.05 MPa accuracy. Data were recorded at increments of 4 cm^−1^ and at time intervals of 4 s for each measurement. Diffusion coefficients are the average of at least six injections of sample.

Calculation of the diffusion coefficients from the absorbance–response curve of the solute is performed using the same procedure described for conventional TDT [19,45,47,48,49], with the assumption that small changes in concentration *C* are directly proportional to variations in absorbance. Diffusion coefficients are extracted from the experimentally measured signal by fitting the response curve to:

(2) $$A\left(t\right)=A_{0}+A_{1}t+A_{2}t^{2}+R\left(C\left(t\right)-C_{0}\right)=A_{0}+A_{1}t+A_{2}t^{2}+\Delta A\sqrt{\frac{t_{R}}{t}\exp\left(-\frac{12D{\left(t-t_{R}\right)}^{2}}{R^{2}t}\right)}$$

where the three first terms *A*_0_ + *A*_1_*t* + *A*_2_*t*^2^ reflect the drift and curvature of the baseline due to small concentration and temperature variations; *t*_R_ is the retention time of the peak, *R* = (∂A/∂C)λ is the sensitivity of the detector (directly dependent on the wavenumber at which the measurements are carried out) and Δ*A* is the peak height relative to the baseline.

### 4.3. Preparation of Experiment

The procedure to select the working wavenumbers for each solute and the experimental protocol have been explained in detail previously [19,45], so only the most relevant points are mentioned below.

**Figure 5:**
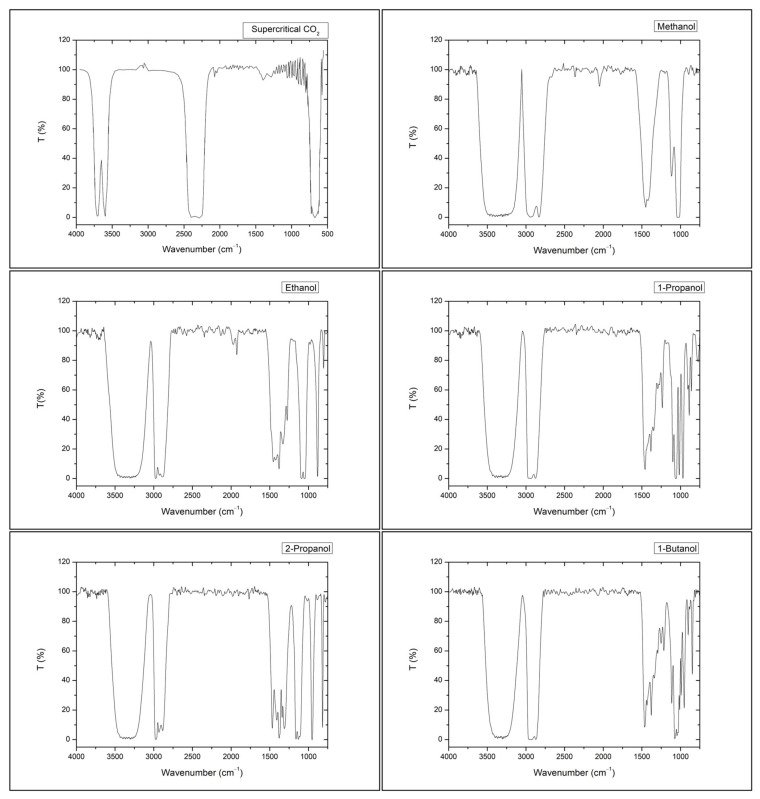
Transmittance infrared spectra of pure solutes. IR spectra for methanol, ethanol, 1-propanol, 2-propanol, and 1-butanol were obtained at 298.15 K and 0.1 MPa. IR spectra for supercritical CO_2_ was obtained at 306.15 K and 10.5 MPa.

Transmittance in the infrared spectra of pure solutes, that is, methanol, ethanol, 1-propanol, 2-propanol, and 1-butanol, at 298.15 K and 0.1 MPa and for supercritical CO_2_, at 306.15 K and 10.5 MPa, were obtained prior to the experiment and are represented in Figure 5.

Supercritical CO_2_ spectra present a region of high absorbance (minimum transmittance) at wavelengths 3500–3800 cm^−1^ that superimposes upon the main stretching frequencies for the alcohol functional group (O–H bond). Even so, these solutes can be identified by their O–H bond bending frequencies [50], although in our experimental measurements, the signal exhibited by this vibration mode is too small to be fitted. Vibration modes, corresponding in general to C–C single bond and C–H bond stretching and bending of these solutes, appear in the supercritical CO_2_ IR transparent regions and originate a signal that corresponds to the dispersion of the pulse of the injected solute in supercritical CO_2_. Thus, from the analysis of the IR spectra in Figure 5, we have followed, with time, the vibration modes identified in Table 4.

## 5. Conclusions

Molecular diffusion coefficients for short-chain alcohols in supercritical CO_2_ were measured by the Taylor dispersion technique in the temperature range of 306.15 to 331.15 K and along the 10.5 MPa isobar.

Diffusion coefficients increased non-linearly with temperature; bulkier and non-linear molecules were less influenced by the temperature effect. The non-linear dependence of diffusion coefficients upon temperature can be explained by the presence of a Widom line within the range of temperatures and pressures studied, a region wherein the supercritical fluid goes through a transition between a liquid-like and gas-like state. The observed behavior is in also agreement with other molecular dynamics and experimental studies for solutes diffusing in supercritical CO_2_.

Dependence on density was analyzed, with diffusion coefficients showing a decrease with an increase of carbon dioxide density, as expected. Various correlation models were assessed to estimate the diffusion coefficients, with the best results obtained by the modified equations by Vaz et al. and He and Yu models in the higher-range densities. In general, classical equations were not able to predict diffusion coefficient behavior within all the conditions studied here, but can be a helpful tool for new systems in which information is completely absent. 

These studies are equally valuable, since they provide qualitative information about the dependence of the *D* coefficients upon the number of methylene groups for short chain alcohols in supercritical CO_2_, helping us to understand the main features of the results and providing transport data necessary to model the diffusion of these solutes in pharmaceutical and industrial applications.

## Figures and Tables

**Table 1:** Measured diffusion coefficients *D* for short chain alcohols in supercritical CO_2_ at pressure *p* = 10.5 MPa, calculated density *ρ* and viscosity *η* [18] for supercritical CO_2_ at different temperatures from 306.15 to 331.15 K.

T/K	*ρ* /kg/m^3^	*η* /(10^−5^ cP)	(*D_Methanol_* ± *S*_D_) /(10^−8^ m^2^ s^−1^)	(*D_Ethanol_* ^a^ ± *S*_D_) /(10^−8^ m^2^ s^−1^)	(*D_1-Propanol_* ± *S*_D_) /(10^−8^ m^2^ s^−1^)	(*D_2-Propanol_* ± *S*_D_) /(10^−8^ m^2^ s^−1^)	(*D_1-Butanol_* ± *S*_D_) /(10^−8^ m^2^ s^−1^)
306.15	752.30	6.37	1.54 ± 0.06	1.49 ± 0.01	1.42 ± 0.06	1.35 ± 0.01	1.25 ± 0.05
311.15	690.79	5.51	1.83 ± 0.05	1.76 ± 0.08		1.61 ± 0.03	
316.15	604.79	4.52	2.01 ± 0.08	1.98 ± 0.05	1.87 ± 0.09	1.75 ± 0.03	1.66 ± 0.07
321.15	489.18	3.49	2.18 ± 0.10	2.10± 0.08	1.95 ± 0.12	1.84± 0.09	1.73 ± 0.08
326.15	393.92	2.85	2.50 ± 0.11	2.48 ± 0.18		2.07 ± 0.12	
331.15	338.03	2.57	3.18 ± 0.14	3.06 ± 0.18	2.66 ± 0.15	2.44 ± 0.12	2.22 ± 0.14

Notes: ^a^ From Ref. [19]; *S*_D_ stands standard deviation of the mean. Standard uncertainties are *u*c (*T*) = 0.01 K and *u*c (*p*) = 0.005 MPa. The expanded uncertainties are *u*c(*D)* ≅ 0.05 × 10^−8^ m^2^ s^−1^ ((level of confidence 0.95).

**Table 2:** The average absolute deviation (*AAD%*) for the predictive equations in the estimation of the diffusion coefficients for short chain alcohols in supercritical CO_2_ at pressure *p* =10.5 MPa and at different temperatures from 306.15 to 331.15 K.

Model		AAD%			
	Methanol	Ethanol	1-Propanol	2-Propanol	1-Butanol
Wilke–Chang ^a^	8.19	4.54	2.98	4.31	3.21
Scheibel ^a^	13.14	9.92	9.10	10.86	10.59
Lusis–Ratcliff ^a^	11.15	5.81	3.49	4.84	3.30
Lai-Tan	4.59	5.28	6.98	8.58	10.46
Sassiat	20.97	14.66	11.78	13.76	11.67
He and Yu	8.63	5.13	3.89	5.27	4.56

Notes: ^a^ Modified models by Vaz et al. [29] The number of points for methanol, ethanol and 2-propanol is 36. The number of points for 1-propanol and 1-butanol is 24.

**Table 4:** Infrared absorption wavenumbers followed for the measurement of diffusion of alcohols in scCO_2_.

Alcohol	Vibration Mode	Absorption Wavenumber
Methanol	C–C single bond	1032 cm^−1^
	C–H bond	2830 cm^−1^
	C–H bond	2943 cm^−1^
Ethanol	C–C single bond	1087 cm^−1^
	C–H bond	2973 cm^−1^
1-Propanol	C–C single bond	1066 cm^−1^
	C–H bond	2963 cm^−1^
2-Propanol	C–C single bond	1112 cm^−1^
	C–H bond	2887 cm^−1^
	C–H bond	2972 cm^−1^
1-Butanol	C–C single bond	1075 cm^−1^
	C–H bond	2934 cm^−1^
	C–H bond	2975 cm^−1^

## Data Availability

The data that support the findings of this study are available from the corresponding author upon reasonable request.
